# Impact of Selected Eicosanoids in Normal and Pathological Pregnancies

**DOI:** 10.3390/jcm12185995

**Published:** 2023-09-15

**Authors:** Małgorzata Szczuko, Justyna Golańska, Joanna Palma, Maciej Ziętek

**Affiliations:** 1 Department of Human Nutrition and Metabolomics, Pomeranian Medical University in Szczecin, W. Broniewskiego 24, 71-460 Szczecin, Poland; 2 Department of Biochemical Sciences, Pomeranian Medical University, 70-204 Szczecin, Poland; joanna.palma@pum.edu.pl; 3 Department of Perinatology, Obstetrics and Gynecology Pomeranian Medical University in Szczecin, Siedlecka 2, 72-010 Police, Poland; maciej.zietek@pum.edu.pl

**Keywords:** pregnancy, gestational diabetes, pre-eclampsia, eicosanoids, prostaglandin, lipoxin, leukotriene

## Abstract

Background: Pregnancy is a physiological state in which the female body undergoes a series of changes and adaptations to provide the best possible conditions for the growth and development of the forming baby. The internal adaptations that take place lead to the production of inflammation, which is necessary for the initial and final stages of pregnancy (embryo implantation and induction of labor). Gestational diabetes mellitus is considered to be the most common pathology during this period. However, many more serious health complications can arise, which include pre-eclampsia, fetal stunting, and preterm labor. The purpose of this study was to analyze the impact of the levels of individual eicosanoids on the course of normal pregnancy and the possibility of pathologies including gestational diabetes and pre-eclampsia. Methods: Sixty-nine pregnant women who were overweight or obese before and during pregnancy were studied. Eicosanoids were extracted as appropriate and then determined using liquid chromatography. The levels of eicosanoids studied in pregnant women differed not only according to the week of pregnancy but also in relation to individual anthropometric and biochemical parameters. Results: There was a significant correlation between being overweight and having a high BMI before pregnancy—as well as biochemical parameters of lipid and carbohydrate profiles—and the occurrence of pathological conditions in pregnancy. Conclusions: Eicosanoids are involved in the pathology of pregnancy associated with the occurrence of gestational diabetes and pre-eclampsia. Salicylic acid may find use in the treatment of pregnant women exposed to both phenomena, as well as in overweight and obese women found before pregnancy. Diets rich in natural salicylates, methods of administration, and pharmacotherapy and dosage need further study. Some of the mediators (lipoxin, prostaglandin and leucotrien) may be new diagnostic markers in pregnancy pathology and intervention pathways in the future.

## 1. Introduction

Pregnancy is a time characterized by waiting for the miracle of birth. The female body experiences physiological adaptive changes as the pregnancy progresses. With the observed growth of the fetus in the following weeks of gestation, there is an increase in the size of the placenta, hypertrophy of the uterine muscle, as well as an increase in the volume of blood plasma and extracellular fluids [1]. The provision of adequate energy, as well as micro- and macronutrients to support pregnancy, is significantly important. Of particular importance are iron, iodine, calcium, magnesium, and potassium [2], as well as folic acid, vitamin D3, B vitamins, and omega-3 fatty acids, the supplementation of which is essential [3,4]. Pregnancy adaptations take place on many levels and involve changes in the anatomical and functional system both within isolated organs and the whole body. The most significant remodeling and reorganization occur within the woman’s reproductive system. The key element is the formation of the placenta from extraembryonic tissues, which act as a link between the mother and child’s body, called the fetal–placental unit [5,6].

### 1.1. Inflammation during Pregnancy

In the early stages of pregnancy, the implantation of the embryo and the invasion of the trophoblast into the myometrium is essential. Proper anchorage of the forming placenta determines the normal course of pregnancy. The placenta is referred to as an accessory endocrine gland in connection with the internal secretion of progesterone, chorionic gonadotropin, and many other hormonally active substances [7]. The peri-implantation period is also a period in which disorders can occur, resulting in complications both in the fetus and in the mother. Among pathologies complicating pregnancy, conditions such as pre-eclampsia (PE), intrauterine growth restriction, gestational diabetes mellitus (GDM), as well as preterm delivery and fetal macrosomia are of clinical importance [8]. A particularly dangerous complication of pregnancy that poses a direct threat to the life and health of the mother and her baby is PE. This condition is defined as the onset of hypertension and proteinuria and may also be accompanied by organ dysfunction involving the liver, kidneys, or central nervous system. PE can progress to an extremely dangerous form called eclampsia, accompanied by generalized tonic–clonic seizures and loss of consciousness [9].

PE is considered a systemic disease that presents with features of vascular endothelial damage, which may affect subsequent abnormalities in the function of other systems, including the cardiovascular system. Although the pathomechanism of PE formation is still under investigation, the involvement of matrix metalloproteinases seems likely. They are regulators in the processes of vascular and myometrial proliferation. Reduced expression of individual metalloproteinases correlates with the process of vasoconstriction of blood vessels, which can result in the occurrence of hypertension and, ultimately, the development of widespread PE and features of failure and insufficient blood supply to the placenta [10,11]. GDM is referred to as a hyperglycemic state, which is first detected during pregnancy. It is recognized that gestational diabetes mellitus is a temporary glucose intolerance caused by failure of the pancreas to secrete insulin by beta cells and insulin resistance of tissues such as subcutaneous, muscle, and liver tissues [12,13]. The diagnosis of GDM is made based on an oral glucose tolerance test or elevated fasting blood glucose [14]. The onset of gestational diabetes correlates with the occurrence of subsequent metabolic complications in both the pregnant woman and the child, while uncompensated gestational diabetes can lead to dangerous consequences, including the induction of fetal birth defects or intrauterine fetal demise and, after delivery, neonatal hyperinsulinemia, hypoglycemia, hyperbilirubinemia, fetal macrosomia, or preterm delivery before the end of the 37th week of pregnancy [12,15].

### 1.2. Pathways of Synthesis in the Inflammatory Process of Arachidonic Acid

Eicosanoids are termed metabolic derivatives of essential unsaturated fatty acids (EFAs), such as arachidonic acid, α-linolenic acid, and linoleic acid. Eicosanoids include prostanoids, leukotrienes (LT), and lipoxins (LX). In addition, prostanoids are divided into smaller groups called prostaglandins (PG), prostacyclins (PGI), and thromboxanes (TX) [16]. Eicosanoids are lipid markers of the body’s physiological and pathological responses (especially with regard to the occurrence of inflammatory reactions), which exhibit different properties depending on how they are synthesized [17]. Depending on the activation of the selected synthesis pathway (cytochrome P450, lipoxygenase, cyclo-oxygenase) individual eicosanoids are formed [17,18]. The eicosanoid pathway involving cyclo-oxygenase leads to the formation of prostanoids (prostacyclins, prostaglandins, and thromboxanes) [19,20,21].

The second eicosanoid pathway associated with the action of lipoxygenase leads to the formation of further eicosanoid derivatives: leukotrienes and lipoxins. Studies indicate [22,23] that in this pathway at least four enzymes are required for arachidonic acid metabolism: 5-LOX, 8-LOX, 12-LOX, and 15-LOX. Through the action of lipoxygenase catalase, arachidonic acid is converted to hydroperoxyeicosatetraenoic acid (HpETE) and then to HETE. Subsequently, the resulting AA metabolites are modified under the influence of activator proteins of individual lipoxygenases and dehydrase, resulting in the formation of leukotrienes and lipoxins [22,23].

The last eicosanoid pathway is the cytochrome P450 pathway. Cytochrome P450 mono-oxygenases convert arachidonic acid into epoxy and hydroxy fatty acids. This occurs through one of three pathways of AA oxidation dependent on reduced triphosphopyridine nucleotide (NADPH). The first of these leads to the formation of fatty epoxy acids (EETs) through surface oxidation. EETs are then metabolized to the corresponding fatty acyl dihydroxyepoxyeicosatrienoic acids under the influence of epoxide hydroxylase [24,25]. The other two arachidonic acid oxidation pathways lead to the production of HETE. PGE2 is also important in the context of fertility and reproduction. In the female reproductive cycle, during ovulation, prostaglandin E2 affects the viscosity of the extracellular matrix so that it produces an environment conducive to fertilization, i.e., optimizing conditions for sperm penetration and its ability to bind to oocytes. In addition, PGE2 is involved in embryo implantation and pregnancy maintenance through the secretion of progesterone by the gestational corpus luteum of the ovary [26].

### 1.3. Role of Eicosanoids 

Lipoxins are among the mediators of inflammation, which act locally in an autocrine or paracrine manner. At the onset of inflammation, lipoxins exert anti-inflammatory effects in nanomole concentrations by overseeing the entry of monocytes and granulocytes (neutrophils and eosinophilia) into the epicenter of inflammation. Lipoxins stimulate macrophages to absorb and remove apoptotic neutrophils at inflammatory sites [27,28].

The level of lipoxins in the body also turns out to be important for the normal menstrual cycle and the various stages of pregnancy development. Lipoxin A4 (LXA4) and its analogs play a significant role here [29]. Having in mind a normal menstrual cycle, it is important that the level of LXA4 in the luteal phase should be elevated and in the follicular phase should be characterized by a noticeable decrease, while the levels of estradiol should be quite the opposite. This is because lipoxin A4 and estradiol compete for the same receptor. In the context of pregnancy, elevated levels of the described lipoxin in the peri-implantation period are inadvisable due to the increased risk of miscarriage. Higher levels of LX4 are necessary for the later stages of pregnancy when changes in the physiology of the reproductive and cardiovascular systems begin to occur. A natural increase in the level of lipoxin A4 should also be observed in the final stages of pregnancy, which affects the reorganization of the blood vessels of the mother-to-be and also enables the separation of the placenta from the uterus in the third period of labor [30]. 

Leukotrienes belong to lipid mediators, whose role is pro-inflammatory. They are synthesized in a paracrine manner and their response depends on the target cell. There are two classes of LT: dihydroxy fatty acid leukotrienes LTB4 and cysteinyl leukotrienes (Cys—LT) [31]. At the site of inflammation, they are formed from mast cells and macrophages, the number of which increases during pregnancy. Individual leukotrienes can promote increased permeability of blood vessels and movement of plasma out of the vascular bed, resulting in edema at the site of inflammation. LTs, due to their pro-inflammatory properties, contribute to some pathological conditions in the body [27,32,33]. 

The purpose of this study was to evaluate the effects of selected eicosanoids in normal and pathological pregnancy on biochemical parameters of carbohydrate, lipid, and liver metabolism.

## 2. Materials and Methods

### 2.1. Characteristics of the Study Group

For the implementation of the study, approval was obtained from the Bioethics Committee of PUM RB: 0012/69/18 dated 18 June 2018.

The criteria for inclusion of patients in the study were as follows:

− confirmation of pregnancy with a positive pregnancy test result (platelet pregnancy tests were used);
− ultrasound examination (GE, Voluson E8 BT19, Boston, MA, USA, 2016);
− The presence of overweight (BMI 25–29.9 kg/m^2^) or obesity (BMI > 30 kg/m^2^) in women before and/or during pregnancy.

All pregnancies were singleton and delivered at or near term (≥38 weeks gestation).

Sixty-nine pregnant women, between 6 and 37 weeks gestation, were included in the study. The mean gestational age was 19.07 ± 8.35 weeks (Table 1). The patients ranged in age from 21 to 45 years. The mean age was 32.21 ± 5.5 years. Anthropometric measurements were taken among the patients, which included height and weight. Height ranged from 1.57 to 1.84 m. The average height was 1.68 ± 0.06 m. Patients’ body weights ranged from 49 to 130 kg. The mean body weight was 84.06 ± 20.79 kg. Based on the anthropometric measurements taken previously, the BMI of each patient was calculated. BMI ranged from 18.44 to 47.86 kg/m^2^. The mean BMI value was 29.75 ± 7.37 kg/m^2^. Among the pregnant women included in the study, blood pressure measurements were taken on the arm twice at 15-min intervals. Ten patients were diagnosed with hypertension based on the flattened measurement criteria: above 140 mmHg systolic pressure and above 90 mmHg diastolic pressure.

#### Division of Patients vs. Groups

The study group of women was divided into two groups: the control group (n = 53) and the pathology group (n = 20), which included pregnancies complicated by gestational diabetes (n = 15) and pre-eclampsia (n = 5). Mean fatty acid mediators were compared between groups (prostaglandin E2, A4 lipoxin 5S, 6R, lipoxin A4 5S, 6R, 15R, leukotriene B4, 16RS HETE), pregnant women’s age, week of pregnancy, pregnant women’s anthropometric parameters (height, pre-pregnancy weight), pregnant women’s BMI before pregnancy, and also their biochemical parameters, which included venous serum concentrations: c-reactive protein (CRP), glucose, alanine aminotransferase (ALT) and aspartate aminotransferase (AST), total cholesterol, HDL fraction cholesterol, LDL fraction cholesterol, triglycerides, gamma-glutamyl transpeptidase (GGTP), insulin, and glycated hemoglobin (HbA1c).

### 2.2. Reagents and Chemicals

The following reagents were used for the study: methanol, boron trifluoride in methanol, acetic acid, chloroform, hexane, acetonitrile, ethyl acetate, hydrochloric acid, 2.6-Di-tert-butyl-4-methylphenol, and sodium chloride. Double-distilled water was also used for the test. The buffers that were used for HPLC analysis were filtered through 0.22 µm nylon filters.

### 2.3. Extraction of Eicosanoids 

In the extraction process, the following were extracted from plasma: prostaglandin E2; lipoxin A4 5S, 6R; lipoxin A4 5S, 6R, 15R; leukotriene B4; and 16RS HETE. To extract the eicosanoids, 0.5 mL of plasma was added to 1 mL of acetonitrile (to precipitate the protein) and 50 μL of internal standard. After a short incubation, the samples were centrifuged using a cooled centrifuge (Eppendorf, 5804R centrifuge). The precipitates were transferred to new collection tubes and 4.5 mL of 1 mM HCl was added. The pH of each sample was equilibrated to 3. The columns were activated with subsequent washes with 3 mL of 100% acetonitrile and 3 mL of 20% acetonitrile in water. The samples were loaded and then washed twice with 3 mL of 20% acetonitrile in water. Eicosanoids were eluted with 1.5 mL of a mixture of methanol and ethyl acetate. dried under vacuum and dissolved in 100 μL of 60% methanol in water with 0.1% acetic acid. The samples were then analyzed using HPLC [34].

### 2.4. HPLC Operating Parameters

HPLC separation was carried out using an Agilent Technologies 1260 liquid chromatograph (Agilent Technologies, Cheadle, UK), which consists of a diode array detector, a degasser column oven, and a bin pump. Agilent ChemStation software (Agilent Technologies, Cheadle, UK) was used for data analysis. Separation was completed using a Thermo Scientific Hypersil BDS C18 column (Agilent Technologies, UK). The temperature of the column oven was 20 °C. A gradient method was used, in which the mobile phase consisted of a mixture of solvents A and B. The content of buffer B in the mobile phase increased from 30% to 98%, and then returned to the initial amount. The DAD detector monitored the peaks by adsorption at 280 nm for leukotriene B4, at 210 nm for prostaglandins E2, 16-HETE, and 16-HETE (the latter two were eluted as a single peak), and at 302 nm for 5S, 6R lipoxin A4, 5S, 6R, and 15R of lipoxin A4. The absorbance spectra of the peaks were analyzed to confirm sample identification. Quantitative analysis was performed using ChemStation software [34].

### 2.5. Statistical Analysis

Analysis of quantitative variables was presented by calculating the mean (avg) and standard deviation (SD) minimums and maximums. The normality of the distribution of variables was tested using the Shapiro–Wilk test. Correlations between quantitative variables were analyzed using Spearman correlation. The significance of correlations is described by the *p*-value. It was assumed that all *p*-values < 0.05 indicated the presence of a statistically significant correlation. The strength of the correlation was evaluated according to the following classification: 0.0| ≤ |r| ≤ 0.2—no correlation; 0.2 < |r| ≤ 0.4—weak correlation; 0.4 < |r| ≤ 0.7—medium correlation; 0.7 < |r| ≤ 0.9—strong correlation; 0.9 < |r| ≤ 1—very strong correlation. The analysis was performed using MedCalc^®^ Statistical Software version 20.218.

## 3. Results

An analysis of biochemical studies using plasma was performed.

### 3.1. Analysis of Total Biochemical Parameters

From the analysis of biochemical blood tests, it was found that the average was not within the reference values specified for pregnant women. The detailed data are shown in Table 2.

### 3.2. Average Levels of Total Fatty Acid Metabolites Analyzed

The levels of selected eicosanoids were analyzed (prostaglandin E2, lipoxin A4 5S, 6R, lipoxin A4 5S, 6R, 15R, leukotriene B4, and 16RS HETE) at different weeks of pregnancy. During the study, prostaglandin E2 levels ranged from 0.018 to 3.678 μg/mL. The average level of this prostaglandin was 0.438 ± 0.590 μg/mL. The level of lipoxin A4 5S, 6R ranged from 0.019 to 3.956 μg/mL. The mean level of this lipoxin was 0.358 ± 0.615 μg/mL. The level of lipoxin A4 5S, 6R, 15R ranged from 0.001 to 0.168 μg/mL. The mean level of this lipoxin was 0.008 ± 0.021 μg/mL. The level of leukotriene B4 ranged from 0.0003 to 0.0766 μg/mL. The mean level of this leukotriene was 0.004 ± 0.0091 μg/mL. The level of the last eicosanoid, i.e., 16RS HETE, ranged from 0.004 to 0.537 μg/mL. The average level was 0.043 ± 0.069 μg/mL. The data are shown in Table 2.

### 3.3. Comparison of the Control Group with the Pathological Group

Different values were found between the groups in height, weight before pregnancy, BMI before pregnancy, glucose, and glycated hemoglobin levels. In contrast, there were no differences in the levels of the fatty acid metabolites tested between the groups. The data are shown in Table 3.

### 3.4. Correlations between Anthropometric and Biochemical Parameters of Pregnant Women and the Level of Total Fatty Acid Metabolites

We analyzed the correlations between the eicosanoids studied (prostaglandin E2, lipoxin A4 5S, 6R, lipoxin A4 5S, 6R, 15R, leukotriene B4, 16RS HETE) and the week of pregnancy, the age of the pregnant woman, and the anthropometric parameters of the pregnant woman (height, pre-pregnancy weight) and BMI before pregnancy. There were numerous correlations at the borderline of significance between the studied eicosanoids and the biochemical parameters of pregnant women. However, several of them were statistically significant. We also found the presence of numerous but weak correlations between the studied eicosanoids and biochemical parameters of pregnant women. Several of them were statistically significant. The data are shown in Table 4.

#### 3.4.1. Correlations between Selected Eicosanoids and Anthropometric and Biochemical Parameters of Pregnant Women in the Control Group

The correlations between the studied eicosanoids and anthropometric parameters were analyzed. A significant correlation of prostaglandin PGE2 with age and LXA4 5S, 6R, 15R with gestational week and pre-pregnancy weight was observed (Table 5). There were numerous mainly weak correlations between the studied eicosanoids and biochemical parameters; in one case, it was statistically significant and concerned the level of lipoxin 5S, 6R, 15R with glycated hemoglobin (Table 5). 

#### 3.4.2. Correlations between Selected Eicosanoids and Anthropometric and Biochemical Parameters of Pregnant Women in the Pathological Group

The correlations between the eicosanoids studied were analyzed, and numerous correlations were found, both weak and of medium strength. Among them, there were two statistically significant ones, namely, the correlations of prostaglandin E2 with body weight and BMI before pregnancy (Table 6). The study of correlations of biochemical measurements showed the presence of numerous weak correlations between the studied eicosanoids and the biochemical parameters of pregnant women. Moreover, three correlations of moderate strength were also found, all statistically significant. These were lipoxin A4 5S, 6R with glycated hemoglobin, leukotriene B4 with triglycerides, and 16RS HETE with LDL cholesterol. The data are shown in Table 6.

### 3.5. Summary of Results 

After testing in the general pregnancy group, the following low but statistically significant correlations were observed:

1. Prostaglandin E2 significantly positively correlated with a pregnant woman’s age, and significantly negatively correlated with her pre-pregnancy weight and BMI;
2. Lipoxin A4 5S, 6R significantly negatively correlated with serum triglyceride levels;
3. Lipoxin A4 5S, 6R, 15R significantly positively correlated with the week of pregnancy, the pregnant woman’s age, and her serum triglyceride level;
4. Leukotriene B4 significantly negatively correlated with a pregnant woman’s pre-pregnancy weight;
5. 16RS HETE significantly positively correlated with the pregnant woman’s serum triglyceride level.

After a comparative analysis of the control group (CG) and pathological group (PG), the following statistically significant correlations were observed:

1. Pregnant women’s height in CG was significantly higher than in PG;
2. The pre-pregnancy weight of women in CG was significantly lower than in PG;
3. BMI of women before pregnancy in CG was significantly lower than in PG;
4. The venous serum glucose level of a woman during pregnancy in CG was significantly lower than that in PG;
5. The venous serum HbA1c level in a woman during pregnancy in CG was significantly lower than PG.

After testing correlations in CG pregnant women, the following low but statistically significant relationships were observed:

1. Prostaglandin E2 significantly positively correlated with the age of pregnant women;
2. Lipoxin A4 5S, 6R, 15R significantly positively correlated with the week of pregnancy and also significantly negatively correlated with the pre-pregnancy weight of women and the level of glycated hemoglobin in pregnant women.

When testing the correlations in the PG of pregnant women, the following moderate statistically significant correlations were observed:

1. Prostaglandin E2 significantly negatively correlated with body weight and BMI before pregnancy;
2. Lipoxin A4 5S, 6R significantly negatively correlated with glycated hemoglobin;
3. Leukotriene B4 significantly positively correlated with triglycerides;
4. 16RS HETE significantly positively correlated with LDL cholesterol.

## 4. Discussion

The comparative self-analysis of the control and pathological groups indicated statistically significant correlations in terms of age, weight before pregnancy, BMI before pregnancy, glucose levels during pregnancy, and glycated hemoglobin levels. This confirms a fact often reported in the literature: that a woman’s age and the occurrence of above-normal body weight and BMI, as well as high glucose and glycated hemoglobin levels, favor the occurrence of pregnancy pathology [35]. Cheng et al. conducted a study on the regenerative properties of prostaglandin E2 in terms of its ability to activate stem cells and angiogenesis, and regulate immune responses. They showed that prostaglandin E2 has a high protective potential for organs against oxidative stress-induced damage and inflammation. Therefore, it is beneficial to maintain it at an optimally elevated level in a state of increased demand [36]. Our research on pregnant women showed a significant positive correlation between the levels of the prostaglandin in question and the age of pregnant women. Age is associated with the development of inflammation and hence the probable increase in the repair potential of eicosanoids with the age of patients through a corresponding increase in its synthesis and levels. The analysis of the results also proved a statistically significant inverse relationship between PGE2 and the body weight and BMI of the patients before pregnancy (both in the pathological group as well as in the overall study group). This may mean that the delicate maintenance of the body’s relative homeostasis in the situation of overweight and obese pregnant patients, by increasing the body’s inflammation, may contribute to the weakening of the regenerative functions of prostaglandin E2, and thus to the occurrence of pregnancy complications [37]. 

Schulte et al., in their study on the relationship between lipid anti-inflammatory molecules and obesity (in the context of bariatric surgery), showed a correlation between excessive body weight and significantly higher levels of pro-inflammatory prostaglandin E2. Such findings may directly influence the onset of diabetes [38]. In the case of our study, an inverse relationship was demonstrated: as body weight increased, a decrease in prostaglandin E2 levels was observed in pregnant women. As is known, the second and third trimesters are diabetogenic periods due to physiological insulin resistance, which could indicate that the body exceeds the buffering limit and is overstimulated, thereby leading to gestational diabetes. However, this thesis should be evaluated on a larger group of obese patients. If confirmed, PGB2 should not be used in pregnant women to predict diabetes due to depleted synthesis.

Undurti N. Das et al. reported the anti-inflammatory nature of lipoxin A4 in their study. They showed that, in order to bring about a cessation of inflammation in the body, a sufficiently high level of lipoxin A4 is important. Otherwise, there will be a continuation of chronic conditions accompanying obesity, type 2 diabetes, rheumatoid arthritis, or hypertension [39].

Lipoxins synthesized in the LOX-15 pathway have various functions in the inflammatory response, including silencing. Bhattacharya et al., in their work, showed a correlation between abnormal levels of glycated hemoglobin in pregnant women and the onset of gestational diabetes, especially in the early stages of pregnancy. They also reported a negative effect of this parameter, which is associated with an increased risk of perinatal complications [40].

In our study, in a group in which pathological conditions of pregnancy occurred, we showed a significant inverse relationship between lipoxin A4 5S, 6R and the level of glycated hemoglobin in the pregnant woman’s serum. With an increase in the level of glycated hemoglobin, there is a simultaneous increase in inflammation in the body and a decrease in the anti-inflammatory lipoxin A4 5S, 6R (due to its utilization). This could have a direct impact on the onset of a pathological condition of pregnancy, such as gestational diabetes mellitus, and similarly to PGE2 reaching its buffering limit. Thus, a decrease in this lipoxin could be a diagnostic parameter in the early stages of gestational diabetes in the future. In our study on the general group, we analyzed two types of the described lipoxin—LXA4 5S, 6R and LXA4 5S, 6R, 15R. The level of the first one significantly inversely correlated with serum triglyceride levels in pregnant women. In contrast, other correlations were shown by analysis of the second type of lipoxin, LXA4 5S, 6R, 15R. In this case, the study proved that the level of administered lipoxin significantly increased in direct proportion to the level of triglycerides in the serum of pregnant women. The reason for the different relationship between the studied lipoxins and the same parameter (triglyceride level) is related to the activation of the synthesis pathway by acetylsalicylic acid (aspirin). After taking a dose of aspirin, the anti-inflammatory LXA4 5S pathway is activated, 6R, 15R. This is referred to as “aspirin-triggered lipoxin” [41]. The second lipoxin studied (LXA4 5S, 6R) is not dependent on acetylsalicylic acid (ASA). In our study, the women did not take ASA-containing preparations, and this may be the reason for the differences in the levels of the two anti-inflammatory lipoxins.

Our study showed a significant positive correlation between aspirin-triggered lipoxin (LXA4 5S, 6R, 15R) and the age of the pregnant woman and the stage of pregnancy. This result is interesting due to the fact that the women were not taking acetylsalicylic acid before the study and it could be used to reduce inflammation in pregnant women, in particular for the presence of gestational diabetes and those who were obese before pregnancy. Johnson et al., in their paper on potential anti-inflammatory therapies for insulin resistance, presented a positive relationship between increased levels of the pro-inflammatory leukotriene B4, body weight, and BMI in humans, as well as the key role of LTB4 in inflammation-induced insulin resistance [42]. However, a different relationship emerged from our study. According to the analysis of the results in the general group, the level of leukotriene B4 was inversely proportional to the above-mentioned factors (of which LTB4 correlated significantly with body weight). The period of pregnancy is a special time when metabolism changes and body weight increases in a woman, including body fat, at an accelerated rate. The results suggest that protective mechanisms during this period are inadequate.

O’Sullivan et al., in their study on the efficacy of omega-3 fatty acid supplementation, noted a simultaneous linear change in triglyceride levels and leukotriene B4. Supplementation with the anti-inflammatory eicosapentaenoic and docosahexaenoic acids resulted in a decrease in pro-inflammatory leukotriene and equally pro-inflammatory triglycerides [43]. An analysis of the results of the pathology group in our study also showed such a statistically significant positive relationship. The increase in the inflammatory marker (LTB4) was due to an increase in serum triglycerides. During pregnancy, a slight increase in triglyceride levels is a physiological adaptation of a woman’s body. However, its excessive increase, along with accompanying other lipid disorders, may contribute to pregnancy complications [44]. 

Our study showed a statistically significant positive correlation between 16-HETE and triglyceride levels in pregnant women in the general group and between 16-HETE and LDL cholesterol levels in the pathological group. It is possible that this results in an increased risk of multi-organ complications (e.g., pre-eclampsia in a pregnant woman) [45]. This relationship has not been encountered in the current literature, so this observation requires further study.

This study used a single point of measurement of mediators in all patients, which is a limitation of this study. The vast majority of women were in the second trimester of pregnancy, which is considered the most stable, therefore the demonstration of numerous relationships in the group of PG women confirms the involvement of mediators in the pathology of pregnancy.

## 5. Conclusions

Women whose body weight and BMI are elevated before pregnancy are at greater risk of developing pathological conditions during pregnancy. The occurrence of gestational diabetes and pre-eclampsia are complex processes with a multifactorial basis, in which the involvement eicosanoids is included. Prostaglandin is related to anthropometric parameters of pregnant women and its share increases in obese women before pregnancy. Week of pregnancy, age of the pregnant woman and triglyceride level are related to the level of salicylic acid-dependent lipoxin. However, Lipoxin A4 5S, 6R is related to the level of triglycerides and HbGA1 in obese people before pregnancy, which creates the possibility of intervention to improve health. Leukotrein and 16RS HETE are related to the lipid metabolism of obese women before pregnancy (trigl and LDL) and, similarly to lipoxin A4 5S, 6R, they may constitute therapeutic targets. Diets rich in natural salicylates, methods of administration, and the pharmacotherapy and dosage need further study.

Some mediators such as LX, PG,h, and LTB4 may be new diagnostic markers in pregnancy pathology and pathways for intervention in the future.

## Figures and Tables

**Table 1:** Characteristics of pregnant women in general.

Parameters	Avg	SD
Week of gestation (week)	19.072	8.351
Age (years)	32.21	5.499
Height (m)	1.682	0.056
Body weight (kg)	84.064	20.786
BMI (kg/m^2^)	29.750	7.371

Avg—average; SD—standard deviation.

**Table 2:** Biochemical parameters of pregnant women in general.

Parameter	Mean	SD	Range of Normal Values *
**Biochemical parameters**			
CRP (mg/L)	6.931	5.127	0.4–20.3
Fasting glucose (mg/dL)	80.256	11.401	75–80
ALT (U/L)	19.254	14.763	2–33
AST (U/L)	17.328	7.980	3–33
Cholesterol Total (mg/dL)	202.835	46.771	176–299
HDL cholesterol (mg/dL)	69.924	14.439	52–87
LDL cholesterol (mg/dL)	125.337	40.615	77–184
Triglycerides (mg/dL)	152.857	66.723	75–382
GGTP (U/L)	13.25	12.249	4–22
Insulin (mU/L)	17.215	17.150	No data available
HbA1c (%)	5.143	0.313	4–6
**Fatty acid derivatives**			
Prostaglandin E2 (μg/mL)	0.43764	0.590	No data available
Lipoxin A4 5S. 6R (μg/mL)	0.35837	0.615	No data available
Lipoxin A4 5S. 6R. 15R (μg/mL)	0.00838	0.021	No data available
Leukotriene B4 (μg/mL)	0.004	0.009	No data available
16RS HETE (μg/mL)	0.04254	0.069	No data available

SD—standard deviation; CRP—C-reactive protein; ALT—Alanine aminotransferase; AST—Aspartate aminotransferase; GGTP—Gamma glutamyl transferase, transpeptidase; HbA1c—Hemoglobin A1c. A HbA1c greater than or equal to 6.5% identifies women with overt diabetes. * range is based on perinatology.com, Reference Values During Pregnancy.

**Table 3:** Comparative analysis of the control group (CG) and pathological (PG) group.

Parameters	SD		Mean		*p*-Value
	CG	PG	CG	PG	
**Biochemical parameters**					
Week of gestation (week)	8.6	8.738	19.207	20.5	0.570
Age (years)	5.808	5.002	31.941	32.368	0.777
Pregnant woman’s height (m)	0.058	0.048	1.688	1.667	0.042 *
Body weight before pregnancy (kg)	19.691	14.355	80.026	98.1	0.0007 *
BMI before pregnancy (kg/m^2^)	6.591	5.914	27.952	36.024	0.00002 *
CRP (mg/L)	5.157	5.329	6.538	8.73	0.130
Glucose (mg/dL)	9.624	14.817	79.038	85.385	0.038 *
ALT (U/L)	15.091	14.406	19.396	19.105	0.942
AST (U/L)	8.428	6.069	17.358	15.789	0.46
Total cholesterol (mg/dL)	42.608	56.225	201.637	208.86	0.563
HDL cholesterol (mg/dL)	13.776	15.663	70.921	63.984	0.073
LDL cholesterol (mg/dL)	36.693	51.235	123.131	136.456	0.227
Triglycerides (mg/dL)	67.943	58.794	147.654	170.892	0.191
GGTP (U/L)	9.743	14.851	11.837	15.722	0.217
Insulin (mU/L)	11.505	14.851	13.467	15.722	0.509
HbA1c (%)	0.307	0.2915	5.108	5.287	0.041 *
**Fatty acid derivatives (µg/mL)**					
Prostaglandin E2	0.6491	0.4463	0.5033	0.2772	0.167
Lipoxin A4 5S, 6R	0.6980	0.8820	0.4227	0.3809	0.837
Lipoxin A4 5S, 6R, 15R	0.0241	0.0375	0.0093	0.0133	0.597
Leukotriene B4	0.0109	0.0171	0.0046	0.0062	0.636
16RS HETE	0.0784	0.1181	0.0452	0.0616	0.505

*—statistically significant difference; SD—standard deviation; CRP—C-reactive protein; ALT—Alanine aminotransferase; AST—Aspartate aminotransferase; GGTP—Gamma glutamyl transferase, transpeptidase; HbA1c—Hemoglobin A1c; CG—control group; PG—pathological group.

**Table 4:** Correlations between individual eicosanoids and anthropometric and biochemical parameters of pregnant women overall.

Parameter		PGE_2_ (µg/mL)	LXA_4_ 5S, 6R (µg/mL)	LXA_4_ 5S, 6R, 15R (µg/mL)	LTB_4_ (µg/mL)	16RS HETE (µg/mL)
**Anthropometric parameters**						
Week of gestation (week)	**R**	0.011	−0.23 *	0.294 **	0.141	0.187
	***p*-value**	0.9283	0.0573	0.0144 **	0.2485	0.1247
Age (years)	**R**	0.347 **	0.133	0.24 **	−0.027	0.071
	***p*-value**	0.0038 **	0.2813	0.0482 **	0.8297	0.5673
Pregnant woman’s height (m)	**R**	0.139	0.037	−0.132	−0.179	−0.028
	***p*-value**	0.27	0.7704	0.2948	0.1542	0.8271
Pre-pregnancy body weight (kg)	**R**	−0.259 **	−0.005	−0.104	−0.26 **	0.128
	***p*-value**	0.0385 **	0.9662	0.4112	0.0377 **	0.3137
BMI before pregnancy (kg/m^2^)	**R**	−0.284 **	−0.017	−0.107	−0.233 *	0.1
	***p*-value**	0.0228 **	0.8929	0.399	0.0644 *	0.4315
**Biochemical parameters**						
**CRP ** **(mg/L)**	**R**	−0.141	−0.175	−0.157	−0.225 *	−0.145
	***p*-value**	0.2656	0.167	0.2158	0.0732	0.2527
**Glucose ** **(mg/dL)**	**R**	0.085	0.227 *	−0.167	−0.19	−0.075
	***p*-value**	0.4956	0.0664	0.181	0.1263	0.5522
**ALT** **(mg/dL)**	**R**	−0.063	−0.109	0.046	−0.127	−0.041
	***p*-value**	0.6137	0.3798	0.7133	0.3051	0.7412
**AST ** **(mg/dL)**	**R**	0.068	−0.114	0.045	0.034	−0.023
	***p*-value**	0.5832	0.3593	0.719	0.7851	0.8557
**Total cholesterol ** **(mg/dL)**	**R**	−0.022	−0.14	0.07	0.121	0.025
	***p*-value**	0.8583	0.2586	0.5724	0.3292	0.8405
**HDL cholesterol ** **(mg/dL)**	**R**	0.111	0.009	−0.022	0.201 *	−0.118
	***p*-value**	0.3721	0.942	0.8603	0.731	0.3426
**LDL Cholesterol ** **(mg/dL)**	**R**	−0.107	−0.146	0.162	−0.02	0.011
	***p*-value**	0.387	0.2379	0.1893	0.8752	0.9275
**Triglycerides ** **(mg/dL)**	**R**	−0.152	−0.252 **	0.25 **	0.142	0.262 **
	***p*-value**	0.2222	0.0415 **	0.0432 **	0.2565	0.0338 **
**GGTP ** **(U/L)**	**R**	−0.172	0.061	−0.018	−0.047	0.055
	***p*-value**	0.1751	0.6312	0.8884	0.7124	0.6638
**Insulin (mU/L)**	**R**	−0.208 *	−0.122	0.017	−0.113	0.173
	***p*-value**	0.0935	0.3281	0.8929	0.366	0.1656
**HbA1c (%)**	**R**	−0.112	−0.138	−0.064	−0.065	0.009
	***p*-value**	0.394	0.2946	0.6262	0.6198	0.9474

PGE_2_—prostaglandin E2; LXA_4_ 5S, 6R—lipoxin A4 5S, 6R; LXA_4_ 5S, 6R, 15R—lipoxin A4 5S, 6R, 15R; LTB_4_—leukotriene B4. **—statistically significant correlation. *—low correlation.

**Table 5:** Correlations between the studied eicosanoids and anthropometric parameters of women in the control group (CG).

Anthropometric Parameters						
Parameters	Correlations	PGE_2_ (µg/mL)	LXA_4_ 5S, 6R (µg/mL)	LXA_4_ 5S, 6R, 15R (µg/mL)	LTB_4_ (µg/mL)	16RS HETE (µg/mL)
Week of gestation (Week)	**R**	0.213 *	0.095	0.367 **	0.238 *	0.165
	***p*-value**	0.1328	0.5093	0.0081 **	0.0923	0.2469
Age (years)	**R**	0.303 **	0.272 *	0.252 *	0.163	0.057
	***p*-value**	0.0327 **	0.0559	0.0775	0.2567	0.6949
Pregnant woman’s height (m)	**R**	−0.152	−0.085	−0.214 *	−0.231 *	0.038
	***p*-value**	0.3017	0.5647	0.1442	0.1144	0.795
Body weight	**R**	−0.271 *	−0.141	−0.288 **	−0.265 *	−0.016
	***p*-value**	0.0657	0.3434	0.0496 **	0.0717	0.9155
Before pregnancy (kg)	**R**	−0.27 *	−0.127	−0.25 *	−0.223 *	−0.047
	***p*-value**	0.0661	0.3951	0.0897	0.1314	0.7526
**Biochemical parameters**						
**CRP (mg/L)**	**R**	−0.231 *	−0.217 *	−0.127	−0.194	−0.252 *
	***p*-value**	0.1106	0.134	0.3842	0.1828	0.081
**Glucose (mg/dL)**	**R**	−0.026	0.039	−0.169	−0.113	0.092
	***p*-value**	0.8577	0.7844	0.2362	0.4314	0.5206
**ALT (mg/dL)**	**R**	−0.078	−0.135	0.011	0.017	−0.094
	***p*-value**	0.5879	0.3465	0.9395	0.9078	0.5095
**AST (mg/dL)**	**R**	0.072	−0.007	0.08	0.104	−0.048
	***p*-value**	0.6138	0.9629	0.5764	0.4695	0.738
**Total cholesterol ** **(mg/dL)**	**R**	0.032	0.088	0.196	0.075	−0.101
	***p*-value**	0.8229	0.5378	0.1689	0.6013	0.4798
**HDL cholesterol (mg/dL)**	**R**	0.124	0.109	0.097	0.033	−0.028
	***p*-value**	0.3843	0.4475	0.4988	0.8164	0.8468
**LDL cholesterol (mg/dL)**	**R**	−0.046	0.003	0.144	−0.005	−0.109
	***p*-value**	0.7505	0.9808	0.313	0.9716	0.4449
**Triglycerides (mg/dL)**	**R**	−0.011	0.128	0.244 *	0.142	0.161
	***p*-value**	0.9415	0.3775	0.0875	0.3265	0.2652
**GGTP (U/L)**	**R**	−0.043	−0.037	0.068	0.107	−0.022
	***p*-value**	0.7703	0.802	0.646	0.4685	0.8823
**Insulin (mU/L)**	**R**	−0.081	0.005	−0.043	−0.069	−0.019
	***p*-value**	0.5781	0.9719	0.7669	0.6337	0.8934
**HbA1c (%)**	**R**	−0.186	−0.055	−0.296 **	−0.231 *	−0.042
	***p*-value**	0.2169	0.715	0.0459 **	0.1231	0.7799

PGE_2_—prostaglandin E2; LXA_4_ 5S, 6R—lipoxin A4 5S, 6R; LXA_4_ 5S, 6R, 15R—lipoxin A4 5S, 6R, 15R; LTB_4_—leukotriene B4. **—statistically significant correlation. *—low correlation.

**Table 6:** Correlations between the studied eicosanoids and anthropometric and biochemical parameters of women in the pathological group (PG).

Anthropometric Parameters						
Parameters	Correlations	PGE_2_ (µg/mL)	LXA_4_ 5S, 6R (µg/mL)	LXA_4_ 5S, 6R, 15R (µg/mL)	LTB_4_ (µg/mL)	16RS HETE (µg/mL)
**Week of gestation (week)**	**R**	−0.224 *	−0.211 *	0.153	0.367 *	−0.092
	***p*-value**	0.3556	0.3857	0.5305	0.1227	0.7083
**Age (years)**	**R**	0.211 *	0.06	0.098	−0.222 *	−0.167
	***p*-value**	0.4013	0.8121	0.698	0.3749	0.5089
**Pregnant woman’s height (m)**	**R**	0.328 *	−0.181	−0.062	−0.113	0.115
	***p*-value**	0.1843	0.4726	0.8063	0.6567	0.6499
**Body weight** **Before pregnancy (kg)**	**R**	−0.56 ***	−0.064	0.423 ***	0.071	0.356 *
	***p*-value**	0.0194 ***	0.8085	0.0904	0.7862	0.1614
**BMI before pregnancy (kg/m^2^)**	**R**	−0.645 ***	−0.019	0.367 *	0.083	0.245 *
	***p*-value**	0.0052 ***	0.9435	0.1474	0.751	0.3432
**Biochemical parameters**						
**CRP (mg/L)**	**R**	−0.267 *	−0.26 *	0.095	0.004	−0.1
	***p*-value**	0.3182	0.331	0.7267	0.9887	0.7113
**Glucose (mg/dL)**	**R**	0.272 *	0.12	0.225 *	0.061	−0.377 *
	***p*-value**	0.2903	0.6455	0.3852	0.8171	0.1362
**ALT (mg/dL)**	**R**	−0.117	−0.031	−0.211 *	−0.389 *	−0.038
	***p*-value**	0.6542	0.9053	0.4152	0.1226	0.8854
**AST (mg/dL)**	**R**	0.07	−0.01	−0.211 *	−0.27 *	0.018
	***p*-value**	0.7882	0.9688	0.4167	0.2953	0.9459
**Total cholesterol ** **(mg/dL)**	**R**	−0.085	−0.295 *	−0.186	0.21 *	0.279 *
	***p*-value**	0.7459	0.2495	0.4748	0.4191	0.2786
**HDL cholesterol (mg/dL)**	**R**	−0.244 *	−0.313 *	−0.169	0.17	−0.246 *
	***p*-value**	0.3454	0.2211	0.5177	0.5135	0.342
**LDL cholesterol (mg/dL)**	**R**	0.087	−0.204 *	−0.193	0.024	0.513 ***
	***p*-value**	0.741	0.432	0.457	0.9263	0.0354 ***
**Triglycerides (mg/dL)**	**R**	−0.288 *	−0.201 *	0.225 *	0.582 ***	0.144
	***p*-value**	0.2629	0.4386	0.385	0.0143 ***	0.5808
**GGTP (U/L)**	**R**	−0.212 *	−0.2	−0.23 *	−0.181	0.007
	***p*-value**	0.4143	0.4423	0.3739	0.4868	0.9798
**Insulin (mU/L)**	**R**	0.304 *	−0.172	−0.149	−0.192	0.045
	***p*-value**	0.2363	0.5091	0.5674	0.4612	0.864
**HbA1c (%)**	**R**	−0.275 *	−0.539 ***	0.131	−0.126	−0.088
	***p*-value**	0.3212	0.038 ***	0.6414	0.6539	0.7564

PGE_2_—prostaglandin E2; LXA_4_ 5S, 6R—lipoxin A4 5S, 6R; LXA_4_ 5S, 6R, 15R—lipoxin A4 5S, 6R, 15R; LTB_4_—leukotriene B4. *—low correlation. ***—moderate correlation.

## Data Availability

Data will be made available upon request.
